# Effect of different chemotherapy schemes on early-stage breast cancer patients with Low HER-2 expression

**DOI:** 10.12669/pjms.39.5.7446

**Published:** 2023

**Authors:** Yurui Xu, Lin Chao, Jianyu Wang, Yonghong Sun, Chen Li

**Affiliations:** 1 Yurui Xu, Department of Breast and Thyroid, Wuxi No.2 People’s, Hospital Affiliated Nanjing Medical University, Wuxi 214002, Jiangsu, China; 2 Lin Chao, Department of Breast and Thyroid, Wuxi No.2 People’s, Hospital Affiliated Nanjing Medical University, Wuxi 214002, Jiangsu, China; 3 Jianyu Wang, Department of Breast and Thyroid, Wuxi No.2 People’s, Hospital Affiliated Nanjing Medical University, Wuxi 214002, Jiangsu, China; 4 Yonghong Sun, Department of Breast and Thyroid, Wuxi No.2 People’s, Hospital Affiliated Nanjing Medical University, Wuxi 214002, Jiangsu, China; 5 Chen Li, Department of Breast and Thyroid, Wuxi No.2 People’s, Hospital Affiliated Nanjing Medical University, Wuxi 214002, Jiangsu, China

**Keywords:** Breast cancer, Adjuvant chemotherapy, Low HER2 expression, Liposome-encapsulated doxorubicin

## Abstract

**Objective:**

To explore the effect of different chemotherapy schemes on the prognosis, immune function and adverse reactions of breast cancer patients with low HER-2 expression after surgery.

**Methods:**

A retrospective analysis was carried out on the clinical data of 60 breast cancer patients with low HER-2 expression in Wuxi No.2 people’s Hospital from January 2018 to December 2019. The enrolled patients were divided into two groups according to the different chemotherapy schemes. Patients in the DC group were treated with polyethylene glycol-coated liposome-encapsulated doxorubicin+cyclophosphamide, and those in the TC group were treated with TC (docetaxel+cyclophosphamide). Further comparison was performed on the difference in prognosis, immune function and adverse reaction between the two groups after different chemotherapy schemes.

**Results:**

After four courses of treatment, the IgG, CD4+ and CD4+/CD8+ values in the DC group after treatment were higher than those before treatment, while the IgG, CD3+ and CD4+values in the TC group after treatment were lower than those before treatment(P<0.05). Meanwhile, the IgG, CD4+ and CD4+/CD8+ values in the DC group were better than those in the TC group after treatment(P<0.05). During the treatment, the adverse reactions of leukopenia, alopecia, nausea and vomiting in the DC group were significantly lower than those in the TC group(P<0.05).

**Conclusion:**

The chemotherapy combination of liposome-encapsulated doxorubicin+cyclophosphamide can significantly improve immune function and greatly reduce the occurrence of adverse reactions in early-stage breast cancer patients with low HER-2 expression after surgery. It has the same effect as docetaxel+cyclophosphamide in improving the prognosis of patients.

## INTRODUCTION

Breast cancer is a common malignant tumor clinically, with the highest incidence in female malignant tumors in the world, which is the leading cause of death in women.1 It is reported that by 2030, there will be approximately 2.64 million new cases of breast cancer and 1.7 million deaths related to breast cancer worldwide.2 In China, breast cancer is the malignant tumor with the highest incidence among women, and its incidence is gradually increasing in women after the age of 30. The incidence of breast cancer in China has increased by >30% in the past decade. However, with the improvement of diagnosis and treatment, the 5-year survival has reached >90% in patients with breast cancer after treatment.3 Surgery is still the major therapeutic option for breast cancer currently.

Postoperative radiotherapy and chemotherapy, endocrine therapy and targeted therapy can significantly improve surgical efficacy and the survival rate of patients. At present, the emergence of ADC anti-HER-2 agents can also benefit some breast cancer with low HER2 expression that was originally classified as triple-negative or Luminal subtypes, which arouses an upsurge of research on breast cancer with low HER2 expression.4 The concept of breast cancer with low HER2 expression has been proposed by the American Society of Clinical Oncology/College of American Pathologists (ASCO/CAP) guidelines.5 According to its definition, breast cancer with low HER2 expression is a new entity defined as HER2 immunohistochemistry (IHC) 1+ or 2+/in situ hybridization (ISH)-negative. As documented by previous research, breast cancer with low HER2 expression can be defined as a new subtype of breast cancer, which has its own biological characteristics and is different from other types in treatment response and prognosis.6 The present study was performed to have a better understanding of the clinical characteristics of breast cancer patients with low HER2 expression, so as to provide a better prognosis and treatment basis for breast cancer with low HER2 expression.

This study carried out statistics and analysis on the immune function and prognosis of early-stage breast cancer patients with low HER-2 expression undergoing different chemotherapy combination and compared the differences in clinical efficacy, adverse reactions, and immune function of patients before and after chemotherapy. Findings in our study are expected to provide evidence for understanding the characteristics of breast cancer with low HER2 expression and guide the selection of an adjuvant chemotherapy scheme for early-stage breast cancer with low HER2 expression.

## METHODS

A retrospective analysis was carried out on the clinical data of 60 breast cancer patients who underwent a radical mastectomy in Wuxi No.2 people’s Hospital from January 2018 to December 2019 were selected and divided into the DC group and the TC group according to different chemotherapy combination.

### Ethical Approval

The study was approved by the Institutional Ethics Committee of Wuxi No.2 people’s Hospital(No.:2023XY-2; date: January 03,2023), and written informed consent was obtained from all participants.

### Inclusion criteria:

- Patients diagnosed with invasive breast cancer.
- Patients with complete clinical and pathological data, with preoperative examination and examination within three months after chemotherapy involving IgA, IgG, IgM and T lymphocyte subsets (CD3+, CD4+ and CD8+) in peripheral blood;
- Patients receiving standard adjuvant treatment after surgery, of which the adjuvant chemotherapy regimen was DC (polyethylene glycol-coated liposome-encapsulated doxorubicin+cyclophosphamide) regimen or TC (docetaxel+cyclophosphamide) regimen;
- Patients with HER2 IHC 1+ or 2+/ISH-negative.

### Exclusion criteria:

- Patients with incomplete clinical data;
- Patients with other malignant tumors;
- Patients receiving neoadjuvant therapy;
- Patients with inflammatory breast cancer, occult breast cancer, metaplastic cancer, male breast cancer, and advanced breast cancer;
- Patients with immune system diseases or acute and chronic infections, taking special drugs, etc.;
- Patients with HER2 IHC 0 or 3+.

Patients who were provided with polyethylene glycol-coated liposome-encapsulated doxorubicin+cyclophosphamide were classified into the DC group, and the chemotherapy regimen was implemented as follows: Day one, intravenous drip of liposome-encapsulated doxorubicin (35 mg/m^2^) + 5% glucose solution (250 mL), and cyclophosphamide (600 mg/m^2^) + 5% glucose solution (500 mL). Patients receiving docetaxel+cyclophosphamide were divided into the TC group according to the following chemotherapy regimen: Day one, intravenous drip of docetaxel (75 mg/m^2^) + 0.9% sodium chloride solution (500 mL), and cyclophosphamide (600 mg/m^2^) + 5% glucose solution (500 mL). The two groups were treated for four consecutive courses of treatment with 21 days as a course of treatment. During chemotherapy, patients were monitored regularly by routine blood tests, liver and kidney function, myocardial enzymogram, lymphocyte subsets, and immune function tests. Meanwhile, liver, kidney, heart protection and other treatments were performed in a timely manner. Anti-infection treatment was given to patients with fever; Those with severe gastrointestinal reactions such as nausea and vomiting were given regular treatment such as preventing nausea, stomach protection and strengthening intravenous nutrition support in a timely manner.

### Outcome measures

Inter-group comparison was conducted to evaluate differences in clinical features, long-term prognosis, immune function and adverse reactions of the two groups. The median follow-up period was 52.5 months. *Immune function indexes:* The peripheral venous blood samples were collected from each patient before the operation and within three months after the end of chemotherapy. An enzyme-linked-immunosorbent assay (ELISA) was used to determine IgA, IgG and IgM in serum. The T lymphocyte subsets (CD3+, CD4+ and CD8+) in peripheral blood before and after treatment were detected by flow cytometry, with the calculation of CD4+/CD8+ at the same time. The post-treatment adverse reactions (e.g., leukopenia, anemia, alopecia and diarrhea, etc.) of the two groups of patients were evaluated according to the National Cancer Institute Common Toxicity Criteria 3.0 (NCICTC3.0). Patients were followed up by telephone and outpatient service after discharge.

### Statistical analysis

SPSS26.0 software was used for statistical analysis. The measurement data were expressed by mean ± standard deviation (*χ̅*±*S*), and inter-group comparison employed t test. The counting data were presented in n (%), and χ² test was used for inter-group comparison. The Kaplan-Meier method was used for survival analysis. P<0.05 meant that the difference was statistically significant.

## RESULTS

There was no significant difference between the two groups in age, tumor diameter, TNM stage, pathological type ER, PR status, HER2 status, Ki67, operation mode, recurrence and metastasis (P>0.05), suggesting comparability between groups. Table-I

**Table-I:** Comparison of general data between the two groups

Clinical characteristics	DC group (n=30)	TC group (n=30)	t/χ^2^ value	P value
Age (years)	57.41±13.23	53.96±12.73	1.235	0.220
Tumor diameter (cm)	2.17±1.26	2.06±1.53	0.276	0.783
***Affected side (n, %)***			1.086	0.297
Left	19(63.33)	15(50.00)		
Right	11(36.67)	15(50.00)		
***TNM stage (n, %)***			1.310	0.519
Stage-I	16(53.33)	13(43.33)		
Stage-II	11(36.67)	11(36.67)		
Stage-III	3(10.00)	6(20.00)		
***Pathological type (n, %)***			0.829	0.842
Invasive lobular carcinoma	4(13.33)	3(10.00)		
Invasive ductal carcinoma	26(86.67)	27(90.00)		
***Operation mode (n, %)***			0.523	0.470
Breast-conserving surgery	3(10.00)	6(20.00)		
Modified radical mastectomy	27(90.00)	24(80.00)		
***Ki67 (n, %)***			5.995	0.050
≤14%	12(40.00)	13(43.33)		
15%-50%	9(30.00)	15(50.00)		
>50%	9(30.00)	2(6.67)		
***HER-2 expression (n, %)***			3.360	0.067
1+	21(70.00)	14(46.67)		
2+	9(30.00)	16(53.33)		
***ER, PR status (n, %)***			0.424	0.809
ER(+)PR(+)	15(50.00)	20(66.66)		
ER(+)PR(-)	7(23.33)	4(13.33)		
ER(-)PR(-)	4(13.33)	1(3.33)		
ER(-)PR(+)	4(13.33)	5(20.00)		
Recurrence or metastasis (n, %)	4(13.33)	2(6.67)	0.185	0.667

**Fig.1:**
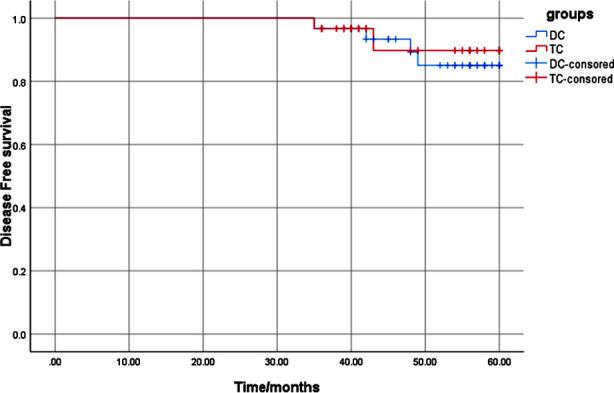
Comparison of disease-free survival between the two groups.

As of December 31, 2022, the median follow-up time was 52.5 (35~60) months, and the mean DFS time was 57.78±0.86 months for the 60 patients with low HER-2 expression in this retrospective study. The two different chemotherapy schemes had no impact on the prognosis of patients. The five years DFS of the DC group was 93.3%, and that of the TC group was 86.7%, without statistical difference between the two groups (χ^2^=0.126, P=0.722; Fig.1).

Before treatment, there was no significant difference in immune function indexes between the two groups (P>0.05). Table-II. After four courses of treatment, the IgG, CD4+ and CD4+/CD8+ values in the DC group after treatment were higher than those before treatment, while the IgG, CD3+ and CD4+values in the TC group after treatment were lower than those before treatment, with statistically significant difference (P<0.05). Meanwhile, inter-group comparison after treatment showed that the IgG, CD4+ and CD4+/CD8+ values in the DC group were better than those in the TC group after treatment, with a statistically significant difference (P<0.05).

**Table-II:** Comparison of immune function between the two groups before and after treatment (*χ̅*±*S*).

Indicators	DC group		TC group	

	Before treatment	After treatment	Before treatment	After treatment
IgA	6.02±2.51	5.82±2.03	6.51±2.06	5.84±1.69
IgG	5.80±0.67	6.31±0.76*	5.94±1.01	5.40±1.04*,^Δ^
IgM	8.00±2.07	8.26±2.24	8.46±2.20	8.51±2.28
CD3+	44.53±8.88	43.32±8.51	44.04±7.52	40.56±7.43*
CD4+	27.23±5.49	29.80±4.28*	28.74±5.13	25.61±4.83*,^Δ^
CD8+	21.12±3.42	20.41±3.41	21.65±3.54	19.07±2.15
CD4+/CD8+	1.29±0.16	1.47±0.12*	1.33±0.23	1.36±0.28^Δ^

***Note:*** Intra-group compariosn before and after treatment, * P<0.05; Inter-group comparison after treatment, ^Δ^P<0.05.

During the treatment, both groups of patients had adverse reactions. Among them, the adverse reactions of leukopenia, alopecia, nausea and vomiting in the DC group were significantly lower than those in the TC group, with a statistically significant difference (P<0.05; Table-III).

**Table-III:** Comparison of adverse reaction rates between the two groups [n (%)].

Toxic and side effects	DC group (n=30)		TC group (n=30)		P

	I~ II	III~IV	I~ II	III~IV	
Leukopenia	8(26.67)	2(6.67)	15(50.00)	4(13.33)	0.001*
Anemia	3(10.00)	0(0)	6(20.00)	3(10.00)	0.052
Constipation	10(33.33)	2(6.67)	12(40.00)	4(13.33)	0.506
Alopecia	1(3.33)	0(0)	5(16.67)	24(80.00)	<0.001*
Liver function damage	4(13.33)	0(0)	7(23.33)	2(6.67)	0.217
Oral mucositis	6(20.00)	5(16.67)	5(16.67)	2(6.67)	0.406
Nausea and vomiting	9(30.00)	1(3.33)	14(46.67)	5(16.67)	0.036*

## DISCUSSION

As anthracycline-free chemotherapy, the TC combination four courses showed a better therapeutic effect than that of the AC combination for early-stage breast cancer, with a median follow-up period of seven years, according to the US Oncology Research Trial 9735.7 Moreover, additional studies in the past have also demonstrated that polyethylene glycol-coated liposome-encapsulated doxorubicin was not inferior to traditional anthracycline antineoplastic drugs, also accompanied by significantly reduced side effects (e.g., nausea, vomiting, alopecia and bone marrow suppression) and obviously alleviated cardiotoxicity.8-10 Nevertheless, so far, there is still no relevant study directly comparing the therapeutic effect of liposome-encapsulated doxorubicin+cyclophosphamide and docetaxel+cyclophosphamide in the treatment of early-stage breast cancer. The present study was performed to compare the two combinations for adjuvant treatment of early-stage breast cancer. After four courses of treatment, the median follow-up was 52.5 months, and there was no difference in PFS between the two groups, suggesting a similar curative effect of the two schemes (P=0.722). Furthermore, the intra-group comparison revealed that the values of IgG, CD4+ and CD4+/CD8+ in the DC group were higher after treatment than those before treatment; and the TC group showed reduced values of IgG, CD3+ and CD4+ after treatment compared with those before treatment (P<0.05). Inter-group comparison after treatment indicated that the values of IgG, CD4+ and CD4+/CD8+ were better in the DC group than those in the TC group (P<0.05). All these results support that the therapeutic effect of DC chemotherapy is not inferior to that of the TC combination scheme.

According to the latest data released by International Agency for Research on Cancer (WHO), the incidence rate of breast cancer (11.7%) exceeded that of lung cancer for the first time in 2020, and about 15% of women in the world die of breast cancer.1 Tumor size, range of invasion, tumor characteristics, and the patient’s own immune function are all influential factors related to the prognosis and quality of life of breast cancer patients. According to prior studies, peripheral T lymphocyte subsets can predict the clinical outcome of breast cancer, and regulatory B cells can exert immunosuppressive function by acting on tumor cells.11-13 The decrease of CD3+T cell count in patients with malignant tumors can significantly affect the initiation, induction and effect of immune response; the reduction of CD4++T cell count can reduce the specific anti-tumor effect; the increase of CD8+T cell count may strengthen cytotoxicity.14-16

Chemotherapy has been recognized to be an effective postoperative auxiliary strategy for breast cancer, which can improve the disease-free survival and survival rate of patients.17 However, it still has many side effects (e.g., nausea, vomiting, alopecia and bone marrow depression), which may cause severe psychological burdens to patients and seriously affect the quality of life of patients after surgery. In view of the multiple postoperative chemotherapy schemes for breast cancer at present, AC and TC are the most frequently used strategies for early-stage breast cancer. The difference between the two combinations is the use of doxorubicin in AC, while the use of docetaxel in TC.18

Simultaneously, the former combination also showed less toxicity and side effects and could improve the immune function of patients, which is safe and effective in clinical application. In addition, it was reported that low HER-2 expression was a risk factor for poor prognosis in early-stage breast cancer patients, with a five year DFS of 84% (95% CI, 80%-88%) and 62% (95% CI, 48%-74%) in patients with HER-2 (1+) and HER-2 (2+) Fish (-), respectively.19,20 While in this study, only six patients had recurrence and metastasis in the enrolled 60 patients with low HER-2 expression. The median follow-up time was 52.5 months, and the mean DFS time was 57.78±0.86 months, showing a good overall prognosis in the studied patients. Our authors speculate that the following two causes may explain a good prognosis in these early-stage breast cancer patients of our study. Firstly, HR-positive patients accounted for a large proportion 91.7% (55/60) in our study; secondly, patients who had previously chosen four courses of DC or TC scheme had clinical characteristics with good prognoses, such as early TNM staging, low Ki67 expression, etc. In this regard, for early-stage breast cancer, the past subtype identification and therapeutic options of breast cancer have brought better benefits to these patients. Further additional classification of low HER-2 expression subtype may not contribute to the change in treatment plans for such patients.

### Limitations

Further subtyping of breast cancer with low HER-2 expression may not be necessary to change the current clinical decisions of patients with early-stage breast cancer. While further research is still required as there was a limited sample size in the present study.

## CONCLUSIONS

Liposome-encapsulated doxorubicin+cyclophosphamide is a safe and effective adjuvant chemotherapy scheme for breast cancer. It can significantly improve immune function and greatly reduce the occurrence of adverse reactions in early-stage breast cancer patients with low HER-2 expression after surgery. Moreover, patients show a satisfactory overall prognosis, suggesting that the treatment scheme has been sufficient under previous clinical decisions.

### Authors’ Contributions

**YX and CL:** Carried out the studies, participated in collecting data, a drafted the manuscript, are responsible and accountable for the accuracy and integrity of the work.

**LC and JW:** Performed the statistical analysis and participated in its design.

**YS:** Participated in acquisition, analysis, or interpretation of data and draft the manuscript.

All authors read and approved the final manuscript.
